# Comparative phylogeography and evolutionary history of schizothoracine fishes in the Changtang Plateau and their implications for the lake level and Pleistocene climate fluctuations

**DOI:** 10.1002/ece3.1890

**Published:** 2016-01-10

**Authors:** Dekui He, Yifeng Chen, Chunlong Liu, Juan Tao, Chengzhi Ding, Yiyu Chen

**Affiliations:** ^1^ The Key Laboratory of Aquatic Biodiversity and ConservationInstitute of HydrobiologyChinese Academy of SciencesWuhanChina

**Keywords:** Demographic history, endorheic lake, glaciation, palaeolake, South Asian monsoon, The Qinghai‐Tibetan Plateau

## Abstract

The water level oscillation of endorheic lakes and extent change of glaciers associated with the Asian monsoon are known as prominent representatives of climatic and environmental events in the Tibetan Plateau during the Quaternary. However, details process in spatial and temporal changes are still debated. We use the schizothoracines as a palaeoclimatic proxy to test two hypotheses concerning the evolution of Quaternary glaciations and lakes of the Changtang Plateau: (1) the Tibetan glaciations generally tended to decrease since the middle Pleistocene; (2) the lakes expansion was driven by summer monsoon rainfall. Based on a wide range‐wide sampling throughout in the Changtang Plateau and its adjacent drainages, we constructed phylogeny and demographic histories of schizothoracines in the Changtang Plateau. Our results showed that the populations of the exorheic rivers and lakes in southern Tibet possessed higher genetic variability, earlier coalescent and expansion times than those of the endorheic lakes in the Changtang Plateau. Population expansions are highly consistent with phases of strong summer monsoon and high lake level during interglacial stages. The maximum growth rate intervals showed three pulses from 64.7 to 54.8, 39.6 to 31.0, and 14.9 to 2.4 kya respectively. The significant positive correlations were found between regional precipitation and genetic diversity, as well as coalescence time of populations in the endorheic lakes. We suggested that the demographic history of the schizothoracines reflects the spatial and temporal changes in climate and lake level, in particular, in regional precipitation gradients associated with changes of the South Asian monsoon, and supports the climatic hypothesis of a general diminishing tend in Tibetan glaciations in the Tibetan Plateau since the middle Pleistocene.

## Introduction

The Qinghai‐Tibetan Plateau (QTP) and adjacent mountains together form the largest and highest mountainous area of the world. Its high topography and vast volume not only has dramatic influences on climate and environment in the area, but also has influence on the northern hemisphere atmospheric circulation (Ruddiman and Kutzbach 1989; An et al. 2001). Although a huge number of studies in geosciences focus on this orogenic system, the important questions concerning its Late Cenozoic uplift and climatic history are still controversial (Royden et al. 2008; Molnar et al. 2010). Historical biogeographic analyses of modern organisms may help to provide a better understanding of palaeogeography and palaeoclimate of this region (He and Chen 2006; Che et al. 2010).

The glaciation throughout the Himalaya and Eastern Tibet is strongly controlled by precipitation changes related to oscillations in the South Asian monsoonal system combined with cooling events (Owen et al. 2005). It is characterized valley and piedmont glacial systems, with some mountain ice caps. Two conflicting views regarding the nature and extent of the Tibetan glaciations had been proposed (Lehmkuhle 1998). Kuhle (e.g., Kuhle 1998) postulated that a unified ice sheet covered nearly the whole Tibetan Plateau during the Last Glacial Maximum (LGM, *c*. 19–26.5 thousand years ago, kya, Clark et al. 2009), but other scientists believed a less extensive glaciation (Zheng et al. 2002; Owen et al. 2008), and supposed that the extent of Tibetan glaciations gradually diminished since the middle Pleistocene (Zheng et al. 2002). The earlier glaciations were generally more extensive than those of the last glacial cycle (Lehmkuhl and Owen 2005; Zhou et al. 2006). For example, the largest glaciation of the Quaternary (Wangkun Glaciation or Naynayxungla Glaciation, *c*. 720–620 kya, MIS 18–16) in QTP occurred in the Middle Pleistocene, followed the glacial extent received progressive diminution over several glacial cycles (Zheng et al. 2002).

The Qinghai‐Tibetan Plateau holds the highest lakes in the world, and the number of lakes is more than one thousand. Total lake area exceed than 41,000 km^2^, which accounts one half of China lake area (Ma et al. 2011). The lakes of the QTP experienced several lake expansion events or even formed a pan‐paleolake during the Quaternary recovered by the evidence recorded of higher lakeshores (Guan et al. 1984; Jia et al. 2001; Shi et al. 2001). The area of palaeolakes during high lake level period exceeded more than four times the present extent, some lakes even exceeded more than 13 times (Li and Zhu 2001). Lake level changes, especially in closed basins, have been usually used as a proxy of monsoon system changes (Lehmkuhle and Haselein 2000; Shi et al. 2001; Yang et al. 2004; Kong et al. 2011; Fan et al. 2012). Changes in the hydrological cycle on the Tibetan lakes are mainly caused by changes of the Asian monsoon system forcing changes in precipitation and evaporation (Benn and Owen 1998; Daut et al. 2010). At a broad spatial scale, the South Asian monsoon (SAM) mainly affects the southern and eastern part of the plateau (Owen et al. 2005), but this influence weakens significantly across the Himalayan and eastern mountain belt, leading to the precipitation decrease sharply from south to north, and from east to west. At a geological temporal scale, the high lake levels are usually associated with stronger summer monsoon climate that coincides with the Northern Hemisphere summer insolation during the interstadial and interglacials in Tibet (Shi et al. 2001; Yang et al. 2004).

Here, we use the schizothoracines as a palaeoenvironmental proxy to test two hypotheses concerning the evolution of Quaternary glaciations and lake‐level fluctuations in the Changtang Plateau (CTP). First, we test the hypothesis of a general diminishing trend in Tibetan glaciations since the middle Pleistocene (Zheng et al. 2002; Lehmkuhl and Owen 2005). If the long‐term diminishing trend in glaciations is prevalent, we would expect that the earlier extensive and harsh glaciers might leave more intensive genetic imprints than the later ones for these fishes, and the populations could have been persistent in large endorheic lakes in the CTP and exorheic rivers of the central QTP during the last glaciations (75.0–15.0 kya, MIS 4 – 2) because of a relatively mild condition. Second, we test the hypothesis of lake expansion driven by summer monsoon precipitation in the Quaternary (Shi et al. 2001). If the asymmetry in monsoon rainfall is a long‐term process, we would expect that the populations of schizothoracines should display a spatial gradient of decreasing genetic diversity from east to west, and expansion of populations should also be consistent with phases of strong summer monsoon and high lake level stages.

**Figure 1:**
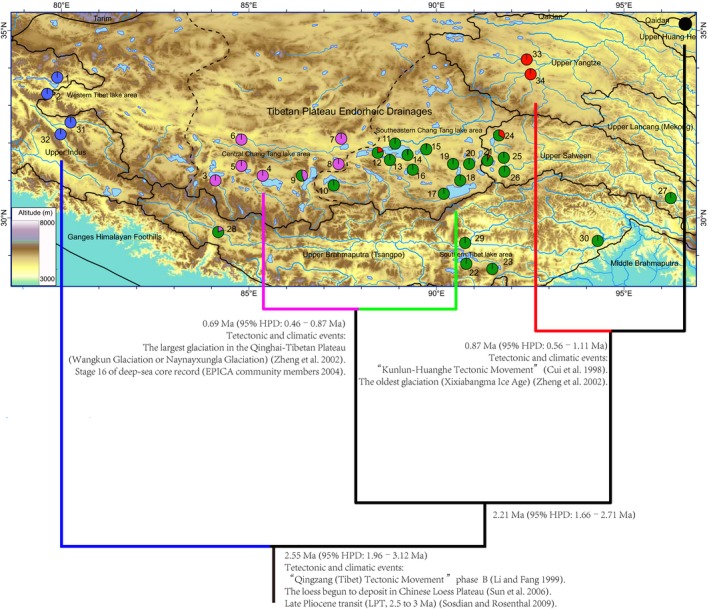
The map of study area and geographical distribution of sampling sites in this study. The cladogram with tentative divergence times for the main mtDNA lineages by Bayesian estimate and major palaeoclimatic events probably associated with cladogenesis are also sketched below relief map. Locality numbers correspond to those in Table 1 (N_loc_). Boundary of main drainage basin is shown by blackline. Pie charts show the frequency distribution of the main haploclades among the populations. Different colors were assigned for each clade recovered by phylogeographic analysis: NET‐N, black; NET‐E, red; WT, blue; CT‐E, green; CT‐C, purple, corresponding to those shown in Figure 2.

To test the hypotheses, we extensively collected the schizothoracine species in the Changtang Plateau and its adjacent drainages (Fig. 1). Most of them were the first sampling record in this region. We then constructed phylogeny of them and demographic histories for each population or species using the mitochondrial cytochrome *b* (cyt *b*) sequences. The evolutionary rate of cyt *b* segment has been calibrated in European cyprinids by a well‐dated isolated event (Zardoya and Doadrio 1999) that could be used to referring the rate in the schizothoracine. Moreover, the mitochondrial gene has a relatively rapid evolutionary rate, is suitable to recover the evolutionary history of the late origin of the highly specialized schizothoracine (Cao et al. 1981; Wang and Chang 2010).

The schizothoracine, a freshwater fish group of Cyprinidae to endemic QTP and high Asia, is particularly suitable to inferring the geological and climatic history in this region (He et al. 2004; He and Chen 2006, 2007; Qi et al. 2012; Li et al. 2013). Three evolutionary adaptive grades, primitive, specialized, and highly specialized, were divided by Cao et al. (1981), based on the extent of modifications of their scales, barbells, and rows of pharyngeal teeth. The vertical distributional patterns of schizothoracines are closely related with their ability of adaptation to the high elevation environmental conditions (Cao et al. 1981). The primary group, including three genera, *Schizothorax, Aspiorhynchus,* and *Schizocypris*, restricts in the periphery of the QTP and adjacent mountain areas, while the highly specialized one, including six genera, *Gymnocypris, Oxygymnocypris*,* Schizopygosis*,* Platypharodon*,* Chuanchia,* and *Herzensteinia*, concentrates on the QTP. In the endorheic drainage of the CTP, only the highly specialized *Gymnocypris* and *Schizopygopsis* could survive in the harsh environments. The dispersal of schizothoracine is limited both by drainage system and elevation, especially in a close basin, where they are unable to descend to a suitable environment at lower altitude during glacial cycles, and experience repeated episodes of population expansion and contraction. Thus, their evolutionary history could thoroughly reflect the geological and related climate events in this region.

## Materials and Methods

### Sample collection, DNA isolation, PCR, and sequencing

The Changtang Plateau, the main part of QTP, is bordered to the east by the Nyainqêntanglha Range, the south by the Gangdese Range, the west by Karakoram Range, the north by the Kunlun Range, and to the northeast by the Hoh Xil and Tanggula Range. The Chantang Plateau stretches approximately 1600 km from east to west, covers 586 000 km^2^ at an average elevation of 5000 m above sea level. We collected a total of 1200 individuals from 34 locations, 16 endorheic lakes in the CTP, two endorheic lakes in the southern QTP, one exorheic lake in the eastern Tibetan, and its adjacent four exorheic river systems throughout in the central Tibetan Plateau (Fig. 1 and Table 1), using gillnets during 2001–2011. These samples were assigned into three genera and eight species and subspecies according to Chen and Cao (2000): *Gymnocypris waddelli* (*N* = 52)*, G. namensis* (44)*, Herzensteinia microcephalus* (57)*, Schizopygosis stoliczkai stoliczkai* (42)*, S. stoliczkai bangongensis* (48)*, S. younghusbandi* (101)*, S. thermalis* (162), and *S*. sp. (*N* = 689), and represent the full distribution range of each species. However, this assignment does not imply to a phylogenetic relationship because there is lack of consensus regarding their taxonomy (Wu and Wu 1992; Chen and Cao 2000).

**Table 1:** Collection localities, drainages, environmental factors, sample size (*N*_ind_), number of haplotype (*N*_hap_), and molecular diversity indexes of the sampled populations of schizothoracines in the Changtang Plateau and its adjacent drainages: haplotype diversity (*h*), nucleotide diversity (*p*), and mean number of pairwise differences (*p*). Localities (*N*_loc_) are numbered consecutively, as shown on the map in Figure 1

Species	N_loc_	Sample locations	Drainages	Latitude N	Longitude E	Altitude (m)	Lake area (km^2^)	Basin area (km^2^)	Precitantion (mm/yr)	N_ind_	N_Hap_	*h* (± SD)	*p* (± SD)	*p* (± SD)
*S. stoliczkai bangongensis*	1	Shankar Shah	Pangong Co	33˚44′	80°17′	4431				32	1	0.0000 ± 0.0000	0.0000 ± 0.0000	0.0000 ± 0.0000
	2	Rutog		33°21′	79°33′	4265				16	2	0.1250 ± 0.1064	0.1250 ± 0.2020	0.0001 ± 0.0002
			Pangong Co			4245	604.0	28110.0	61	48	2	0.0417 ± 0.0395	0.0417 ± 0.1099	0.0000 ± 0.0001
*S. *spp.	3	Tarok Co	Tarok Co	31°03′	83°59′	4572	486.6	6929.4	200	35	12	0.8471 ± 0.0389	1.6874 ± 1.0126	0.0015 ± 0.0010
	4	Zhari Nam Co	Zhari Nam Co	30°53′	85°05′	4617	996.9	15433.2	250	102	11	0.3661 ± 0.0610	0.9814 ± 0.6707	0.0009 ± 0.0007
	5	Dagwa Co	Dagwa Co	31°04′	84°51′	4628	114.5	2419.6	200‐300	20	3	0.2789 ± 0.1235	0.6684 ± 0.5348	0.0006 ± 0.0005
	6	Dong Co	Dong Co	32°07′	84°54′	4416	87.7	5538.0	150‐200	66	3	0.0890 ± 0.0477	0.0900 ± 0.1633	0.0001 ± 0.0002
	7	Tuksitugar Co	Tuksitugar Co	31°49′	86°48′	4469	244.7	10885.3	200	62	5	0.1846 ± 0.0659	0.1914 ± 0.2458	0.0002 ± 0.0002
	8	Kang Co	Kang Co	31°34′	87°21′	4611	88.0	1560.0	200‐300	29	3	0.3596 ± 0.1031	0.3793 ± 0.3703	0.0003 ± 0.0003
	9	Tangra Yum Co	Tangra Yum Co	31°05′	86°29′	4528	835.3	8219.7	300	43	6	0.6102 ± 0.0479	4.3610 ± 2.1980	0.0038 ± 0.0021
	10	Ngangtse Co	Ngangtse Co	30°55′	87°06′	4684	461.5	7132.0	300	37	7	0.7327 ± 0.0504	1.2132 ± 0.7911	0.0011 ± 0.0008
	11	Tsachu Tsangpo	Siling Co	32°02′	89°09′	4560				32	6	0.4315 ± 0.1039	1.5020 ± 0.9292	0.0013 ± 0.0009
	12	Chargut Co		31°48′	88°22′	4558				20	10	0.8000 ± 0.0886	18.4474 ± 8.5402	0.0162 ± 0.0084
	13	Ali Tsangpo		31°44′	88°32′	4566				36	7	0.5968 ± 0.0827	2.4079 ± 1.3386	0.0021 ± 0.0013
	14	Bochu Tsangpo		31°46′	89°21′	4554				62	10	0.6864 ± 0.0574	3.8334 ± 1.9540	0.0034 ± 0.0019
			Siling Co			4544	1628.0	42194.0	290‐321	150	20	0.6328 ± 0.0423	5.2489 ± 2.5518	0.0046 ± 0.0025
	15	Pongok Co	Pongok Co	31°32′	89°44′	4532	140.0	1391.0	308	54	7	0.5584 ± 0.0521	2.4605 ± 1.3516	0.0022 ± 0.0013
	16	Gomang Co	Gomang Co	31°18′	89°10′	4629	97.0	945.0	300	25	4	0.4167 ± 0.1152	0.5133 ± 0.4479	0.0005 ± 0.0004
*G. namensis*	17	Ragyam	Nam Co	30°54′	90°15′	4729				25	9	0.7767 ± 0.0721	2.2267 ± 1.2701	0.0020 ± 0.0012
		Namtso		30°55′	90°56′	4729				19	7	0.7310 ± 0.0857	1.9649 ± 1.1628	0.0017 ± 0.0011
	18		Nam Co			4729	1961.5	8648.5	487	44	12	0.7452 ± 0.0557	2.0793 ± 1.1854	0.0018 ± 0.0012
*S*. sp.	19	Pam Co	Pam Co	31°25′	90°55′	4534	135.7	1020.3	300‐400	28	5	0.4286 ± 0.1083	2.6825 ± 1.4716	0.0024 ± 0.0014
	20	Bam Co	Bam Co	31°19′	90°30′	4565	190.9	4839.2	300‐400	38	5	0.3713 ± 0.0954	1.8222 ± 1.0722	0.0016 ± 0.0010
*S. thermalis*	21	Co Nqion	Co Nqion	31°28′	91°27′	4515	61.3	1019.7	400‐500	40	4	0.3154 ± 0.0913	7.2077 ± 3.4496	0.0063 ± 0.0034
*G. waddelli*	22	Trigu Co	Trigu Co	28°43′	91°38′	4610	56.8	1180.2	300‐400	21	6	0.7524 ± 0.0610	2.0667 ± 1.2048	0.0018 ± 0.0012
	23	Yamdrok Co	Yamdrok Co	29°11′	90°36′	4446	638.0	5422.0	373	31	12	0.7871 ± 0.0715	2.7140 ± 1.4814	0.0024 ± 0.0014
			Southern Tibet							52	14	0.7896 ± 0.0453	2.5030 ± 1.3714	0.0022 ± 0.0013
*S. thermalis*	24	Amdo Tsonak Co	Amdo Tsonak Co	32°07′	91°24′	4590	182.4	3199.6	400	69	13	0.7298 ± 0.0450	22.7528 ± 10.1380	0.0199 ± 0.0099
	25	Nag	Upper Salween	31°26′	91°59′	4483				22	8	0.8658 ± 0.0426	3.8918 ± 2.0298	0.0034 ± 0.0020
	26	Yu Chu		31°12′	91°48′	4704				19	5	0.7310 ± 0.0805	2.9240 ± 1.6034	0.0026 ± 0.0016
	27	Baxoi		30°03′	96°57′	3207				12	5	0.8333 ± 0.0691	3.0152 ± 1.6901	0.0026 ± 0.0017
			Upper Salween							53	11	0.8171 ± 0.0329	3.3512 ± 1.7464	0.0029 ± 0.0017
*S. younghusbandi younghusbandi*	28	Zhongba	Tsangpo	29°46′	83°59′	4570				27	14	0.9288 ± 0.0303	4.5926 ± 2.3271	0.0040 ± 0.0023
	29	Chushul		29°19′	90°41′	3596				40	18	0.9333 ± 0.0215	4.1910 ± 2.1264	0.0037 ± 0.0021
	30	Miling		29°13′	94°12′	2930				39	9	0.8124 ± 0.0380	3.2551 ± 1.7138	0.0029 ± 0.0017
			Tsangpo							106	29	0.9235 ± 0.0126	4.1535 ± 2.0824	0.0036 ± 0.0020
*S. stoliczkai stoliczkai*	31	Gar	Upper Indus	32°31′	80°09′	4290				35	15	0.8655 ± 0.0475	3.3008 ± 1.7384	0.0029 ± 0.0017
	32	Gunsa		32°00′	80°08′	4318				7	3	0.5238 ± 0.2086	1.9048 ± 1.2281	0.0017 ± 0.0012
			Upper Indus							42	16	0.8211 ± 0.0543	3.0441 ± 1.6176	0.0027 ± 0.0016
*H. microcephalus*	33	Tuotuo He	Upper Yangzte	34°13′	92°26′	4542				18	8	0.8889 ± 0.0416	2.5686 ± 1.4447	0.0023 ± 0.0014
	34	Dri Chu		33°52′	92°22′	4569				39	15	0.9069 ± 0.0245	3.6842 ± 1.9038	0.0033 ± 0.0019
			Upper Yangzte							57	17	0.9048 ± 0.0186	3.4712 ± 1.7972	0.0030 ± 0.0017
			Clade CT							1021	121	0.8665 ± 0.0071	5.9169 ± 2.8270	0.0052 ± 0.0027
			Subclade CT‐E							683	81	0.8470 ± 0.0119	3.3341 ± 1.7139	0.0029 ± 0.0017
			Subclade CT‐C							338	30	0.4046 ± 0.0343	0.9964 ± 0.6741	0.0009 ± 0.0007
			Clade WT							90	18	0.6916 ± 0.0472	3.9965 ± 2.0169	0.0035 ± 0.0020
			Subclade NET‐E							89	20	0.8527 ± 0.0268	3.6762 ± 1.8774	0.0032 ± 0.0018
			All samples							1200	149	0.9007 ± 0.0055	17.3429 ± 7.713	0.0015 ± 0.0075

Each individual was subsampled (fin clip or muscle tissue) into 95% ethanol and stored at −20°C. Total genomic DNA was isolated by standard phenol–chloroform extraction protocol (Sambrook et al. 1989). The mitochondrial gene *b* gene was amplified and sequencing adopted from He and Chen (2006). The sequences have been deposited in the GenBank library under the Accession Nos. KC782576 – KC782724 (Appendix S1).

### Nucleotide polymorphism

Electropherograms were visually checked using Chromas 2.22 Technelysium Pty Ltd, South Brisbane QLD, Australia and aligned using Clustal X 2.0 (Larkin et al. 2007). Haplotype diversity (*h*), nucleotide diversity (*π*) and mean number of pairwise differences (*p*) were calculated for each location, basin and for every significant haploclade derived from the phylogenetic analysis results, using Arlequin 3.5.1 (Excoffier and Lischer 2010).

### Analysis of factors influencing genetic diversity

Lakes area, catchments basin area, precipitation and altitude varied substantially in each sampled sites. We used nonparametric Spearman rank correlation to determine the degree of correlation between environmental factors and genetic variability of populations, performing separate analyses for each genetic metric (*h*,* π*, and *p*) using PAST 2.17c (Hammer et al. 2001).

### Phylogenetic analyses

The phylogenetic analyses were performed under maximum likelihood (ML) and Bayesian inferences, using PhyML 3.0 (Guindon et al. 2010) and MrBayes v3.2.3 (Ronquist et al. 2012) respectively. The best‐fit model of nucleotide substitution (GTR + G + I), was selected among 88 alternative models by AIC using jModelTest 2.1.4 (Darriba et al. 2012). The model included unequal base frequencies (A = 0.2618, C = 0.2666, G = 0.1601, T = 0.3115), six substitution categories (AC = 2.3425, AG = 165.9202, AT = 3.6248, CG = 9.5903, CT = 39.8612, GT = 1.0000), a proportion of invariable sites (I = 0.3870) and a rate heterogeneity among sites following a gamma distribution with value 0.4950. For ML analysis, tree topology search was conducted under schemes GTR nucleotide substitution model, optimize equilibrium frequencies, estimated proportion of invariable sites and gamma distribution parameter, nearest‐neighbor interchange (NNI), and BioNJ as initial tree. The node support was assessed by the nonparametric bootstrap method with 1000 replicates. For Bayesian analyses, two independent runs with four Metropolis‐coupled Monte‐Carlo Markov Chains each were simultaneously conducted with GTR + G + I substitution model, 20 million generations, and 1/1000 sample frequency. The first 4000 trees (standard deviation of split frequencies is below 0.01 as the convergence diagnostics) was discarded as burn‐in phase. Nodal support was assessed by calculating the mean posterior probabilities (BBP) values of each node of the resulting consensus tree after burn‐in.

Considering similar morphological characters (Wu and Wu 1992), geographical distribution and low genetic differentiation (He and Chen 2007), the phylogenetic analyses were conducted by the haplotypes without considering taxonomy. In addition, nine haplotypes of *S. pylzovi,* representing 42 individuals and eight sites in the northeastern QTP (the upper Yellow River, Tsaidam Basin and Hexi Corridor) (He and Chen 2007), were also included in phylogenetic analyses in order to obtain a comprehensive phylogenetic relationship. Three species of genus *Gymnocypris* distributed in the northeastern QTP, *G. prezwaslskii, G. eckloni, and G. potanini* were designated as outgroup. Phylogenetic relationships among haplotypes were also inferred by statistical parsimony procedure for phylogenetic network estimations (Templeton et al. 1992) by using the software TCS 1.2.1 (Clement et al. 2000), with a 95% criterion for a parsimonious connection.

### Demographic history analyses

Tajima's *D* (Tajima 1989) and Fu's *F*_*S*_ (Fu 1997) tests of neutrality were calculated to detect range expansions. The significance of both values was calculated from 1000 simulated samples using a coalescent algorithm. Mismatch distribution analysis was performed to distinguish between models invoking past exponential growth versus historical population stasis (Rogers and Harpending 1992; Excoffier 2004). The goodness‐of‐fit was evaluated using parametric bootstrapping with the sum of squared deviations (*SSD*). A significant sum of squared deviations (*SSD*;* P *≤* *0.05) indicates a departure from the null model of population expansion. Neutrality tests and mismatch distribution analyses were performed for each of the populations and phylogroups inferred by phylogenetic analyses using Arlequin 3.5.1 (Excoffier and Lischer 2010). To avoid lineage‐specific effects, haplotypes resulting from secondary contact were excluded in demographic history analyses at basin level.

### Coalescent analysis and divergence date estimations

We used the BEAST program to create Bayesian Skyline plots for each lake, drainages and lineage. BEAUti 1.8.2 was used to generate the input file for the Bayesian Markov chain Monte Carlo (MCMC) analysis of molecular sequences implemented in Beast v1.8.2 (Drummond and Rambaut 2007; Drummond et al. 2015a,b). The model GTR + G was selected by the AIC using jModelTest 2.1.4 (Darriba et al. 2012) for 149 haplotypes within ingroup. A relaxed uncorrelated lognormal molecular clock model (Drummond et al. 2006) was firstly applied, in order to appraise the clock‐like behavior of the data. However, since sampled marginal posterior probability distribution of both standard deviation and coefficient of variation of substitution rates among tree branches was close to zero (ucld.stdev < 0.5), so a simpler strict clock model was used in subsequent analyses. We adopted an evolutionary rate 1.82% per million years for the cyt *b* sequences of genus *Gymnocypris* (He et al. 2004), which was calibrated by a reliable geological dating in the upper Yellow River (Li et al. 1996). This evolutionary rate is also well consistent with 1.86% per million years for cyt *b* gene of *Gymnocypris* species (Duan et al. 2009) calibrated by European cyprinids (Zardoya and Doadrio 1999). All simulations were run for 200–400 million generations, sampling every 1000 of 2–4 independent runs, well above what is minimally required Skyline parameters for acceptable effective sample sizes values (i.e., >100) (Drummond and Rambaut 2007). Tracer 1.6 (Rambaut et al. 2013) was used to check for their convergence, determine burn‐in value, and assess effective sample size of relevant parameters. All runs were combined in LogCombiner 1.8.2 (Rambaut and Drummond 2015a) with burn‐in set. A maximum clade credibility tree was obtained with TreeAnnotator 1.8.2 (Rambaut and Drummond 2015b). Branch‐specific rates and lengths were visualized with FigTree 1.4.2 (Rambaut 2014).

The timing of divergence with 95% highest posterior density (HPD) between lineages was also tentatively estimated using BEAST under a GTR + I + G model with a strict clock assumption and Yule tree prior (Drummond and Rambaut 2007). Two independent Markov Chain Monte Carlo analyses were run for 40 million generations each, sampling every 1,000 generations. The summary statistics and tree were generated by combined trees using LogCombiner v1.8.2 and TreeAnnotator v1.8.2 with 25% burn‐in of each run.

### Population growth rate calculations

We calculated the population growth rate per millennia with the population growth curves generated by BEAST (see above). We also estimated the time intervals when population began growth and the growth rate reached at the fastest (Table 1). Each Skyline plot consisted of 100–500 smoothed data points, at ≈ 1.0 kya intervals. We chose the exponential growth equation (Gignoux et al. 2011) for this analysis: *r *= ln (*N*_*t*2_/*N*_*t*1_)/*t*, where “*r*” represents the population instantaneous growth rate per millennia, “*N*_*t*1_” and “*N*_*t*2_”are the estimated population size at time *t*_1_ and *t*_2_ respectively. A negative *r* indicates that population size begins to decrease since time t_1_.

## Results

### Patterns of genetic diversity

We obtained 1140 bp of the mitochondrial cytochrome *b* gene and identified 149 unique haplotypes from 1200 individuals. Numbers of detected haplotypes for each species/subspecies or sites are listed in Table 1. One hundred twenty‐one haplotypes were private recorded at the drainage basin level (*n *=* *121, 81%). It is noticeable that more than half of shared haplotypes (15, 54%) occurred among species/subspecies with the closely drainage basins (Appendix S1).

The most frequent haplotype (H49) was shared among 260 individuals (260) and occurred in all seven sampled drainage basins in the central CTP (sample 3–9), followed by the haplotype H101,which was shared among 245 individuals and existed in twelve drainage basins in southeast CTP (sample 8, 11–19, 21) and the upper Salween river.

Estimates of nucleotide (*p*) and haplotype diversity (*h*) for each sampled sites and drainage basins are given in Table 1. Haplotype diversity averaged over all species was 0.9007 ± 0.0055 (mean ± SD), with a range from 0.5238 ± 0.2086 to 0.9333 ± 0.0215 for exorheic rivers and 0.0 to 0.8471 ± 0.0389 for endorheic lakes respectively. Nucleotide diversity (*π*) averaged over all samples was 0.0015 ± 0.0075, ranging from 0.0 and 0.0199 ± 0.0099 for *S. stoliczkai bangongensis* from Shankar Shah at Pangong Co and *S. thermali* from lake Amdo Tsonak Co at the upper Salween river respectively. The high nucleotide diversity was observed those sites located at the transition zone between the exorheic and endorheic drainages or between the central and southeastern endorheic lakes, for example Siling Co, Amdo Tsonak Co, Co Nqion and Tangra Yum Co. In general, populations of the exorheic rivers and lakes in the southern QTP possessed higher genetic diversities than those of endorheic lakes in the CTP, as well as the southeastern lakes had higher than the western and central ones within the CTP.

### Analysis of factors influencing genetic diversity

Nonparametric Spearman rank correlation showed that haplotype diversity, average number of nucleotide differences and nucleotide diversity were significantly related to precipitation of drainage basin (Table 2), but that were not significantly affected by an altitude or area, although we observed the expected positive correlation between lake area and genetic diversity.

**Table 2:** Nonparametric spearman correlation coefficients between environmental factors and indicators of genetic diversity (haplotype diversity *h,* average number of nucleotide differences *p* and nucleotide diversity *π*), and the time to most recent common ancestor (TMRCA) in the Changtang Plateau

Genetic indices	Spearman rank correlation coefficients			
	Altitude	Lake area	Basin area	Precipitation
*h*	−0.0825	0.3088	−0.1351	0.7878**
*p*	−0.0211	0.1702	−0.0298	0.8081**
*π*	0.3491	0.1982	−0.1821	0.4993*
TMRCA	0.1558	0.3127	−0.0918	0.7776**

*0.01 < *P *≤* *0.05, **P ≤* *0.01.

### Phylogeographical structures

**Figure 2:**
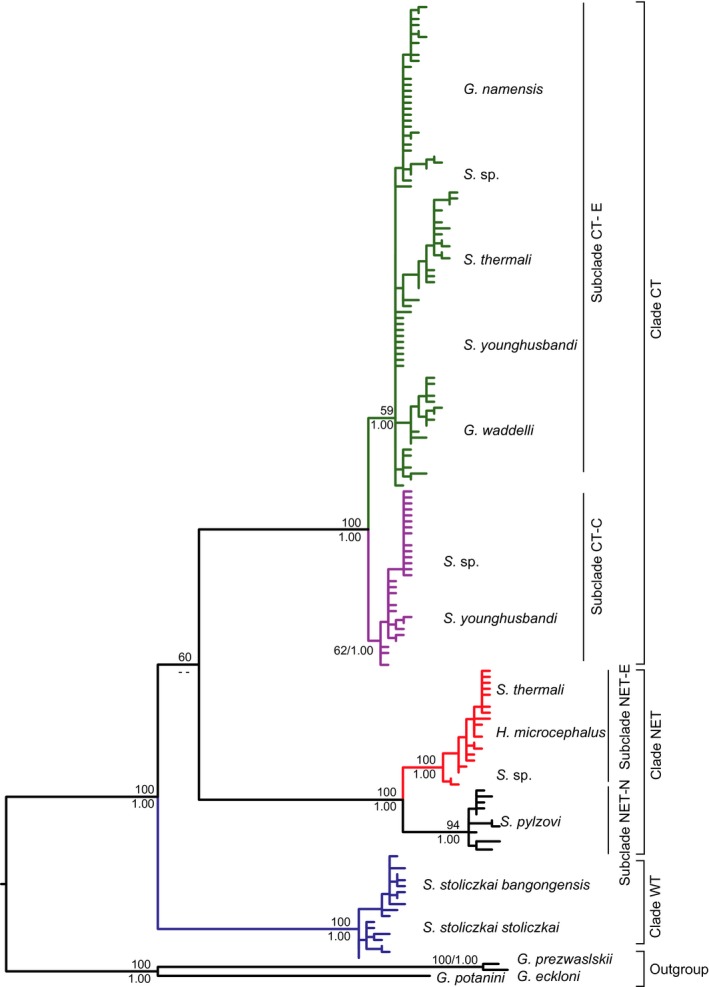
Maximum likelihood tree for the schizothoracine fishes in the Changtang Plateau and its adjacent drainages based on mtDNA cytochrome *b* haplotypes. Clade credibility values of major lineages are given for nodes with bootstrap support for ML (above branch) and posterior probability for Bayesian inferences (below branch). Major clades referred to in the text are listed to the right. Different colors were assigned for each clade: NET‐N, black; NET‐E, red; WT, blue; CT‐E, green; CT‐C, purple.

The ML tree is shown in Figure 2. The phylogenetic analyses yielded a phylogram with three well‐supported clades, each defined largely by geography (Fig. 1). The first clade (referred to as clade WT, the western QTP) included 18 haplotypes of *S. stoliczkai*, and geographically restricted to the upper Indus river and Pangong Co in the western QTP (samples 1, 2 and 31, 32). The second clade (referred to as clade NET, the northeastern QTP) was composed of *S. pylzovi* and 20 haplotypes that limited in the upper Yangtze River (*H. microcephalus,* samples 33, 34) and its adjacent lakes (*S. thermali* and *S*. sp.) such as Siling Co, Pongok Co, Co Nqion and Amdo Tsonak Co (samples 12, 15, 21 and 24). The third clade (referred to as clade CT, the central QTP) was widespread throughout lakes in the CTP (*G. namensis* and *S*. sp.), two lakes in the southern QTP (*G. waddelli*), the Tsangpo (*S. younghusbandi*) and upper Salween rivers (*S. thermali*). Two subclades within clade CT (referred to as subclade CT‐E and subclade CT‐C, Fig. 2) also showed a clear geographic association (Fig. 1). Subclade CT‐C was restricted in the central lakes in the CTP (*S*. sp., samples 3–9) and one sample site in the upper Tsangpo (*S. younghusbandi,* sample 28), subclade CT‐E was found in the lakes in the southeastern CTP (*G. namensis* and *S*. sp., samples 10–21) and the southern QTP (*G. waddelli,* samples 22–23), as well as two exorheic rivers, the upper Salwen (*S. thermali,* samples 24–27) and Tsangpo rivers (*S. younghusbandi,* samples 28–30). Two well‐supported subclade within clade NET (referred to as subclade NET‐N and subclade NET‐E, Fig. 2) were also distinguished. The subclade NET‐N, consisting of eight haplotypes of *S. pylzovi*, limited to the upper Yellow River and interior catchments in the northern QTP (e.g., Tsaidam basin and Hexi Corridor). The subclade NET‐E was restricted to in the upper Yangtze River and its adjacent lakes in the eastern CTP (Fig. 1). The topology obtained from Bayesian inferences was similar to ML tree and supported three clades and fours subclades, but relationships among three clades presented in differences.

**Figure 3:**
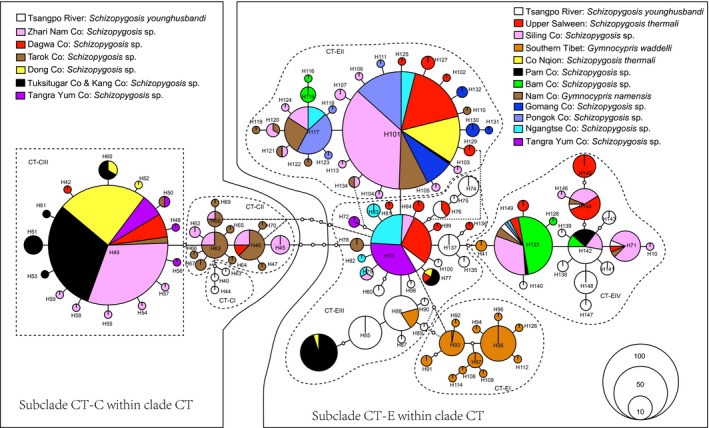
Statistical 95% parsimony network CT generated by TCS based on mtDNA cytochrome *b* haplotypes for the schizothoracine fishes in the Changtang Plateau and its adjacent drainages. Numbers in the networks represent haplotype designations and the areas of the circles are proportional to haplotype frequency; open dots represent missing intermediate haplotypes.

The statistical parsimony analysis revealed three distinct separate networks (Networks ET, WT and CT; Appendix S2 and Fig. 3) clearly corresponding to the three clades recovered by the phylogenetic analyses. Within network CT, two regionally defined haplogroups could be distinguished (Fig. 3), corresponding to phylogenetic subclade CT‐E and subclade CT‐C. Both main subclades showed signs of further substructuring with a clear geographic association. Substructure CT‐CI was only observed in the upper Tsangpo River (samples 28), CT‐CII was restricted in three southern lakes (samples 3–5) of the central CTP, and CT‐CIII was found in all of seven endorheic lakes in the central CTP, and showed a star‐like topology. Instead, substructure CT‐EI was found exclusively in two endorheic lakes in the southern QTP (Yamdok Co and Trigu Co), CT‐EII was restricted in the endorheic lakes in the southeastern CTP and the upper Salween River, also showed a star‐like topology. Finally, substructures CT‐EIII and CT‐EIV were widespread throughout the endorheic lakes of the southeastern CTP, southern QTP, upper Salween and Tsangpo rivers.

The divergence times for the main mtDNA lineages estimated Bayesian was sketched in Figure 1. Of the species examined, the initial split occurred during the early Pliocene and later Pleistocene (*c*. 2.55 Ma, 95% HPD: 1.96–3.12 Ma, and the separation of clade ENT from the remaining clades occurred *c*. 2.21 Ma (95% HPD: 1.66–2.71 Ma). The estimated divergence times between subclade CT‐C and subclade CT‐E, subclade NET‐N and subclade NET‐E were tentatively dated back to *c*. 0.69 Ma (95% HPD: 0.46–0.87 Ma), 0.87 Ma (95% HPD: 0.56–1.11 Ma) respectively.

### Demographic history

At drainage basins level, all samples except for Tangra Yum Co, Nam Co, Co Nqion and Amdo Tsonak Co, the values of *SSD* and Harpending's Raggedness index *r* received by goodness‐of‐fit tests were not significant (*P *>* *0.05), indicating that the model of sudden expansion cannot be rejected. This was also reflected by Tajima's *D* and Fu's *Fs* which, albeit highly variable among samples, tends to be negative (except Tangra Yum Co, Pam Co and Bam Co) even significant (*P *<* *0.05) (Table 3), indicating departure from neutrality. For the lineages revealed by phylogenetic analyses, Tajima's *D* and Fu's *Fs* statistics were significant and negative (*P *<* *0.01) for subclade CT‐E and CT‐C indicating that a neutral model is rejected. The values of *SSD* (0.0002) and Harpending's Raggedness index *r* (0.1250) were also not significant for subclade NET‐E, clade WT and subclade CT‐E, while there was a contradictory result between values of *SSD* and Raggedness index *r* for subclade CT‐C. Similar phenomena were also found in Nam Co and Amdo Tsonak Co, implying that intraspecific polymorphism of these regions might have been mainly influenced by historical demography and not selection.

**Table 3:** Results of neutrality tests and demographic analyses. Sample size (*N*_ind_), number of haplotype (*N*_hap_), Tajima's *D* (*D*), Fu's *Fs* (*Fs*), sum of squared deviations (SSD), Raggedness index (*r*), and mismatch distribution parameter *τ* are listed

Drainage/Haplogroup	*N*_ind_	*N*_Hap_	Neutrality tests		Demographic analyses		*τ* (95% Confidence interval, CI)
			*D*	*Fs*	SSD	*r*	
Pangong Co	48	2	−1.1069	−1.6022*	0.0000	0.8420	3.000 (0.574–3.000)
Tarok Co	35	12	−1.3305	−5.9526**	0.0023	0.0577	1.799 (1.029–2.654)
Dagwa Co	20	3	−1.1787	0.3503	0.0405	0.4674	3.000 (0.430–3.172)
Zhari Nam Co	102	11	−1.3956	−5.2035*	0.0367	0.3128	3.000 (0.000–4.078)
Dong Co	66	3	−1.3151*	−2.6932**	0.0001	0.684	3.000 (0.453–3.000)
Kang Co	29	3	−0.5281	−0.4865	0.0048	0.1932	0.461 (0.031–1.051)
Tuksitugar Co	62	5	−1.6811*	−4.7493**	0.0011	0.4360	3.000 (0.369–3.500)
Tangra Yum Co	43	6	1.3900	4.4712	0.2045*	0.3706*	9.188 (0.145–95.188)
Ngangtse Co	37	7	−1.2984	−1.7347	0.0095	0.1098	1.188 (0.684–1.974)
Siling Co	145	16	−0.8331	−3.6829	0.0778	0.1769	6.211 (0.004–92.211)
Pongok Co	53	6	−1.6931*	−1.7737	0.0151	0.1512	0.742 (0.344–1.238)
Gomang Co	25	4	−0.8789	−1.3032	0.0007	0.1310	0.514 (0.008–1.096)
Nam Co	44	12	−1.3750	−3.8676*	0.2418**	0.0394	0.348 (0.031–0.891)
Bam Co	38	5	−0.4410	1.3637	0.0472	0.3065	3.000 (0.334–3.889)
Pam Co	28	5	0.1412	2.2234	0.1251	0.4179	3.00 (0.000–3.900)0
Co Nqion	37	3	−1.6431*	0.4572	0.0387*	0.6857	3.000 (0.461–3.211)
Amdo Tsonak Co	46	9	−1.4750	−2.0409	0.3311**	0.1146	0.000 (0.000–0.000)
Southern Tibetan Lakes	52	14	−1.1622	−4.1822*	0.0279	0.1014	3.301 (0.340–6.035)
Tsangpo	106	29	−1.1619	−11.1647**	0.0042	0.0127	5.332 (1.750–8.852)
Upper Salween	53	11	0.0421	−0.4054	0.0396	0.0599	6.900 (0.137–59.900)
Upper Indus	42	16	−1.3415	−5.8307*	0.0079	0.0127	7.574 (0.063–26.879)
Upper Yangtze	57	18	−0.6240	−5.3347*	0.0060	0.0205	3.020 (0.672–8.156)
Clade WT	90	18	−0.4672	−2.5350	0.0778	0.1381	8.057 (0.008–17.189)
Subclade NET‐E	89	20	−0.5723	−4.6373	0.0129	0.0330	5.859 (0.807–10.051)
Clade CT	1021	112	−1.5385*	−24.2308**	0.0164	0.0344	9.824 (2.184–15.830)
Subclade CT‐C	338	30	−2.0977**	−28.9367**	0.2061**	0.2392	0.000 (0.000–0.600)
Subclade CT‐E	683	82	−1.9092**	−25.3436**	0.0134	0.0320	5.416 (0.857−9.670)

*P ≤* *0.05; **P ≤* *0.01.

The time to most recent common ancestor (TMRCA) estimates for populations at drainage basin level are given in Table 4. Samples from lakes in the eastern CTP and southern QTP, and the exorheic rivers, coalescence occurred between *c*. 353.90 – 36.0 kya. For populations from the lakes in the central and western CTP, coalescence occurred during the last glacial cycle (*c*. 112.0–6.4 kya). In general, the coalescence times of populations in eastern lakes were earlier than that of west part of plateau. We also observed the significant positive correlation (Spearman *r *=* *0.7776, *P *<* *0.001) between precipitation and estimated coalescent times of endorheic lakes (Table 2).

**Table 4:** Population growth rates calculated from skyline plots and the time to most recent common ancestor (TMRCA) for populations at drainage basin level and four clades of the schizotoracine fishes in the Changtang Plateau

Drainages/clades	Growth began, kya	95% CI for start of growth, kya	Fastest growth interval, kya	Time at maximum growth rate, kya	Maximum growth rate	Mean TMRCA, kya	95% HPD interval, kya
Pangong Co	7.3	15.2–0.56	4.4–0.9	2.4	0.6753	6.4	22.1–0
Tarok Co	81.0	121.7–52.0	22.9–7.6	14.9	0.0121	112.0	216.7–33.5
Zhari Nam Co	34.2	46.7–21.3	9.1–2.3	5.7	0.3542	109.9	198.3–19.0
Dagwa Co	35.0	62.5–17.1	11.1–2.1	4.3	0.0730	83.5	207.8–6.1
Dong Co	9.1	19.7–1.9	5.4–1.7	3.8	0.6352	9.6	31.8–0.0
Tuksitugar Co	17.2	35.3–6.6	11.5–5.4	8.9	0.4265	15.1	42.5–1.2
Kang Co	18.1	19.2–4.7	11.0–1.9	5.4	0.0901	24.9	75.2–0.6
Tangra Yum Co	19.6	36.1–8.1	9.1–2.5	5.8	0.1426	353.9	576.7–130.2
Ngangtse Co	78.3	116.5–43.6	43.4–6.5	11.6	0.2634	145.5	309.5–27.5
Siling Co	92.3	99.2–62.3	53.5–27.4	36.7	0.0635	220.2	396.4–69.5
	14.0	82.9–6.9	7.2–3.1	6.1	0.5521		
Pongok Co	11.8	28.9–5.1	5.9–2.0	4.3	0.4635	197.4	371.5–32.7
Gomang Co	31.5	57.5–11.7	16.9–1.9	6.8	0.1152	36.0	95.3–2.1
Nam Co	94.1	105.3–83.1	18.4–5.8	11.5	0.0646	227.6	391.8–75.4
Bam Co	10.5	26.4–2.7	5.0–0.9	3.1	0.2154	282.6	495.1–84.1
Pam Co						258.0	462.83–113.4
Co Nqion						191.1	361.3–37.2
Amdo Tsonak Co	189.9	–	13.1–5.6	9.1	0.0245	252.0	440.8–69.7
Southern Tibetan lakes	148.1	170.1–112.2	103.2–23.9	8.9	0.0163	195.4	407.6–89.4
Tsangpo	146.8	181.1–113.7	86.2–31.3	61.4	0.0192	246.7	401.7–103.5
Upper Salween	130.2	146.4–98.7	68.4–17.4	31.0	0.0113	232.0	397.3–86.3
Upper Indus	141.2	165.9–116.5	71.5–36.2	56.2	0.0383	277.7	467.4–110.2
Upper Yangzte	143.2	172.4–114.2	104.1–25.0	64.7	0.0149	206.4	340.1–95.7
Clade WT	177.3	200.6–138.4	80.7–17.6	39.6	0.0183	321.5	526.3–136.0
Subclade NET‐E	132.6	162.3–107.6	39.6–4.6	8.3	0.0570	202.2	360.2–87.1
Subclade CT‐C	36.6	56.9–23.9	8.0–2.5	4.5	0.7659	111.4	209.3–22.6
Subclade CT‐E	171.0	186.8–125.4	103.2–40.1	54.8	0.0145	285.2	466.1–146.9
	17.9	30.4–13.0	14.1–12.5	12.0	0.6477		

The BSPs showed three lineages, subclade NET‐E, subclade CT‐E and clade WT, population expansions began at a transit period from MIS 6 to MIS 5 (*c*. 177.3–132.6 kya, Table 4 and Appendix S3). In contrast, the subcalde CT‐C that was mainly consist of samples from lakes in the central and western CTP, skyline began to grow rapidly by 36.6 kya (CI: 56.9–23.9 kya) (Table 4 and Appendix S3). At drainages basin level, except for three lakes (Pam Co, Co Nqion and Amdo Tsonak Co) that locate in a transition zone between the exorheic and endorheic drainages in the eastern edge of CTP, all populations experienced fast population growth phases since 104.1 kya (Table 4).

### Population growth rate

**Figure 4:**
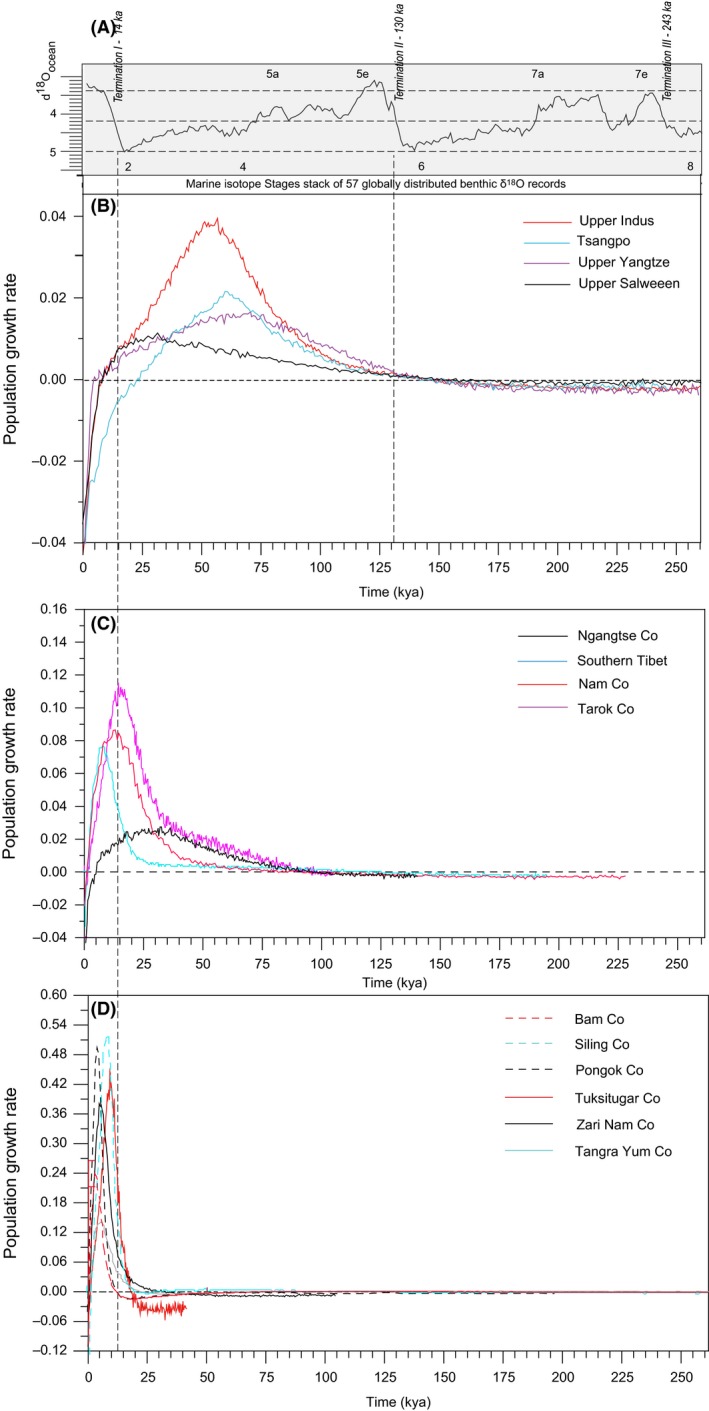
Instantaneous population growth rate plotting through time at drainage basin level from mtDNA cytochrome *b* sequences. The x axis represents time in 10^3^ years, the y axis is estimated population instantaneous growth rate per millennia calculated by the exponential growth equation (*r *= ln (*N*_*t*2_/*N*_*t*1_)/*t*). (A) Marine isotope Stages stack of 57 globally distributed benthic *δ* ^18^O records (adapted from Cohen and Gibbard, Global chronostratigraphical correlation table for the last 2.7 million years, http://www.quaternary.stratigraphy.org.uk/correlation/chart.html); (B) Four exorheic river systems; (C, D) endorheic lakes.

For the four exorheic rivers and endorheic lakes in the southern QTP, the population growth rate inferred by the BSPs showed an accordant tendency. The increases of all populations were preceded by a long period of slow decrease, following a rapidly growing period beginning around termination II (*c*. 130 kya) (Fig. 4B and Table 4). The maximum growth rates of the exorheic Indus, Tsangpo, and Yangtze took place during transmit period from MIS 4 to MIS 3 (*c*. 64.7–54.8 kya), whereas the upper Salween occurred in late MIS 3 (*c*. 31.0 kya), hereafter, the population growth rate decreased rapidly, even become negative following the Last Glacial Maximum. For the large endorheic lakes in the CTP, the fastest growth intervals occurred during the late last interglaciation corresponding to late MIS 5 (*c*. 130–74 kya) (e.g., Nam Co, Ngangtse Co, Tarok Co and Siling Co) and interstadial MIS 3 (e.g., Zhari Nam Co) (Fig. 4D and Table 4). In contrast, the remaining lakes displayed a rapidly growth after LGM.

**Figure 5:**
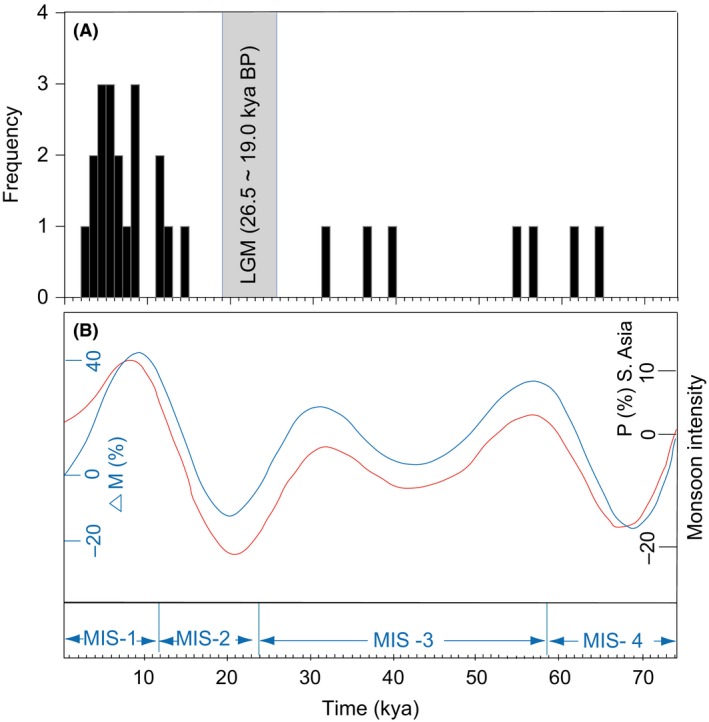
(A) Frequency histograms of maximum growth rates intervals of populations for each drainage basin and four significant haploclades versus time per millennia. (B) Simulated monsoon pressure index (ΔM percentage, solid blue line) for the Indian Ocean and simulated changes in precipitation (ΔP percentage, red line) in southern Asia (adapted from Owen et al. 2008).

For the 20 expansion populations at basin level, the maximum growth rate interval occurred within past 64.0 kya. A histogram of the maximum growth rate intervals showed three pulses from 63.4 to 54.8 (early MIS 3), 39.6 to 31.0 (late MIS 3), and 14.9 to 2.4 kya (post LGM) respectively (Fig. 5).

## Discussion

### Phylogeographical structure and taxonomic implications

Our results revealed a distinct geographical structure across the CTP. The examined schizothoracines were clustered into three well‐supported clades, each defined largely by geography. Except for two species, *S. stoliczkai* and *S. pylzovi*, the remaining species did not form a monophyly. The share haplotypes existed among species, even between genera. The haplotype sharing was a common phenomenon in schizothoracines (Qi et al. 2006; He and Chen 2007; Duan et al. 2009). However, the sharing events in this case mainly occurred in the sites located at eastern margin of CTP (e.g., Siling Co, Pongok Co, Co Nqion) and the lake Tangra Yum Co. These sites are either located at the transition zone between the exorheic and endorheic drainages, or between the central and southeastern endorheic lakes in the CTP. The tentative estimated divergence times for these populations were consistent with the high lake‐level and warm climate phases (160.0 ± 8.5 kya for the upper Yangtze and Siling Co, 52.5 ± 24.0 kya for the upper Yangtze and Salween, 31.0 ± 18.5 kya for the upper Salween and Tsangpo, and 4.8 ± 2.0 kya for the Siling Co and upper Salween, respectively) (Jia et al. 2001; Fan et al. 2012), suggesting that a secondary contact and admixture resulted from population expansion originating from different genetic lineages during interglacial or after postglacial stages (reviewed in Hewitt 2011).

### Demographic history and glacial extent changes

Our analyses reveal that the populations of lakes in the southern QTP and the exorheic rivers possessed higher genetic variability and earlier coalescent times than those of endorheic lakes in the CTP, and similar patterns are observed along the endorheic lakes from east to west in the CTP (Fig. 4 and Table 4). This phylogeographic pattern was consistent with the general trend of diminishing of the glacial extent and weakening of the South Asian monsoon since the middle Pleistocene in the QTP due to continued mountain uplift (Owen et al. 2005). Moreover, the tentatively estimated divergence time of two lineages (subclade CT‐E and CT‐C) far predated the LGM even the penultimate glaciation (*c*. 190–130 kya, MIS 6, Riss glaciation) (see Fig. 2), indicating that those fishes persisted in the CTP lakes during the penultimate glaciation and thereafter LGM, thus rejecting the hypothesis of an extensive inland ice sheet during the last glacial cycle (Kuhle 1998).

The estimates of the growth rate suggested that population expansion began approximately 146.8–130.2 kya (Table 3 and Fig. 4B) in the exorheic rivers and lakes in the southern QTP, corresponding to the transit from MIS 6 to MIS 5e. Similar results were also found in *Schizothorax o'connori* distributed in the Tsangpo River (*c*. 145.4–92.7 kya, He and Chen 2009), and the plateau avian species (*c*. 0.17–0.15 Ma, Qu et al. 2010; Zhan et al. 2011), indicating that the extensive penultimate glaciation had a substantial impact on the population size of exorheic rivers. The montane species distributed in the exorheic rivers species could survive in the same region by altitudinal shifts or minor range changes (Hewitt 2000); therefore only earlier extensive and harsh glaciers could result in a significant decrease the effective population size, for example the Wangkun Glaciation and the penultimate glaciation, while the later mild glacier advances occurring in the last glacial cycle had apparently little effect on their population size (Fig. 4). In contrast, the populations growth of lakes in the CTP occurred at the later relatively warm period after the MIS 6, for example the last interglaciation (*c*. 130–74 kya, MIS 5), interstadial of the last glaciation (MIS 3) and retreat period of LGM, indicating that the later mild glacial advances (MIS 4, mid‐MIS 3, and MIS 2) could also have had a significant effect on population size, even resulted in a bottleneck or elimination, owing to its inability to retreat to areas in lower altitude during glacial expansion. It is evident for the population of shallow and small lakes in the central and western CTP, the most recent glacial advance (MIS 2, LGM) produced a marked population bottleneck indicated by more recent coalescence time and low genetic diversity than populations that inhabit the large and deep lakes.

### Genetic diversity spatial trend and the east‐west asymmetry in monsoon precipitation

The correlation analyses revealed a significant positive correlation between precipitation and genetic diversity, which was the same with coalescence time of populations in the endorheic lakes, suggesting that precipitation associated with changes of the monsoon system play an important role in shaping the genetic pattern and demography of fish populations in the QTP. Lake expansion in the QTP is mainly induced by the increase of precipitation, the glacier melting water, and decrease of potential evapotranspiration (Daut et al. 2010). Precipitation in Tibet is mainly derived from the SAM (Benn and Owen 1998). In broad spatial scale, precipitation shows a significant decrease from south to north and from east to west. In arid areas of the QTP, the precipitation and extent of glaciation have become restricted due to weakening summer monsoon. Thus, the western lakes have apparent shrinkage compared to those of the eastern CTP, especially small and shallow lakes with little or no glacier meltwater supplying, resulting in low genetic diversity even bottlenecks.

### Population expansion and lake‐level fluctuation driving by monsoon intensity changes

The maximum growth rate intervals of populations at basin level occurred within past 63.4 kya, and the frequency distribution formed three discrete phases: 63.7–54.8, 39.6–31.0, and 14.9–2.4 kya respectively. It was broadly coincident with the warm climate and high lake levels periods in the QTP (Fan et al. 2012). MIS 3 (*c*. 59.0–26.1 kya) was a relatively warm interstadial in the Last Glacial cycle. The temperature recovered from Guliya ice core in the northwest Tibetan Plateau was abnormally high (Yao et al. 1997), especially during 40–30 kya BP (corresponding to late MIS 3), it almost reached the extent of the Last Interglaciation (Shi et al. 2001). Large freshwater lakes and high lake levels widely occurred in QTP during this period, for example high lake levels of Lake Qinghai occurred in 60–54 and ~40 kya, Gahai (63–55, ~30 kya), Nam Co (~40 kya) and Siling Co (30–27 kya) (Fan et al. 2012). Intervals at post LGM were consistent with two high lake levels periods during 19–15 and 13–11 kya, reflecting increase of glacial meltwater during deglaciation phases following LGM (Kong et al. 2007).

The histogram showed that maximum growth rate intervals were consistent with the period phase of Asian monsoon enhancement (Fig. 5; Owen et al. 2008), supporting the hypothesis of lake expansion was driven by summer monsoon rainfall (Shi et al. 2001). Strong summer monsoon might bring more precipitation to plateau interior during interglacial stages, for example MIS 1, 3, and 5, and resulted in lake levels rising, available habitat enlarging and growing‐season prolonging for fish. Warm climate also promoted fish growth, and then increased the effective population size. In contrast, weak summer monsoon resulted in significant decrease of precipitation and a low level, or even possible desiccation, for these small and shallow lakes during glacial period (Kong et al. 2007).

### Genetic differentiation and palaeoenvironmental history in the Changtang Plateau and its adjacent drainages

Of the species examined, the initial divergence among lineages tentatively dated back to the later Pliocene and early Pleistocene (*c*. 2.55 Ma, 95% HPD: 1.96–3.12 Ma), a period associated with intensification of regional tectonic activities and global climate distinct cooling events during the late Pliocene transit (LPT, 3–2.5 Ma) (Sosdian and Rosenthal 2009). Similar result was observed in the blood pheasant (*Ithaginis cruentus*) in the eastern edge of QTP (*c*. 2.5 Ma, Zhan et al. 2011). The Qinghai‐Tibetan Plateau experienced violet tectonic events during the late Pliocene, also known as the ‘the Qingzang Movement’, which caused large geomorphologic and tectonic reconfigurations (Li and Fang 1999). For example, the Co Ngoin Lake (sample 22), a fault basin situated in the fault zone between the Tanggula Range and the Nyainqêntanglha Range in the eastern margin of the CTP, formed about 2.8 Ma and began to receive lacustrine deposits around 2.6 Ma (Shen et al. 2004). In the western Tibetan Plateau, the upper Tsangpo River and its tributaries flowed westward into the Zanda, Kirong, Dinggyê, and Gamba Paleolakes before 2.5 Ma, after that time, they turned to the eastward (Li 2010). These tectonic events were highly concordant with the tentatively estimated divergence times and probably related to the schizothoracines lineages split among the upper Indus (clade WT, *c*. 2.55 Ma), upper Yangtze (clade ET, *c*. 2.21 Ma), central and southeastern CTP respectively.

On the other hand, the climate of Northern Hemisphere has undergone profound changes during the period from the mid‐Pliocene to the late Pleistocene, which led to the development of large‐scale Northern Hemisphere ice sheets and attributed directly to global cooling (Shackleton et al. 1984). Strengthening of the East Asian winter monsoon and the corresponding weakening of the summer monsoon appears to be a manifestation of global cooling after 3.6 Ma, especially when ice sheets expanded in the Northern Hemisphere at about 2.7 Ma (An et al. 2001; Haug et al. 2005). The various climate proxies has showed distinct cooling events associated with the late Pliocene transit (LPT, 2.5–3 Ma), for example intra‐tooth isotopic patterns of mammalian tooth enamel from the northeastern margin of the QTP revealed significant changes in the seasonal patterns of diet and climate after ~2–3 Ma. Prior to ~2–3 Ma, there was little or no seasonal variation in herbivores' diets and all herbivores fed on C3 vegetation year around. After that time, the data show a significant seasonal variation in the diets of horses and bovids, ranging from a pure C3 to a mixed C3/C4 diet, indicating an enhanced monsoon climate since about 2–3 Ma (Biasatti et al. 2010). The paleoclimatic and paleoecological history of Chinese Loess Plateau (CLP), which have been widely accepted as a valid geologic archive for documenting the history and variability of the East Asian monsoon and related climate (e.g., An et al. 2001; Guo et al. 2002), showed that the CLP experienced dramatic cooling and drying (greater seasonality of precipitation) trends during the 4–2.6 Ma (Bai et al. 2009). The aeolian ‘red clay’ were transformed into loess deposits in CLP around 2.6 Ma which reflected that the global climate was dramatic shifting from dry and warm to dry and cold (Sun et al. 2006). These widely distributed observations can be interpreted as signaling an environmental response to a major phase of Himalaya‐Tibetan plateau uplift during late Pliocene. All of these climatic changes had significant ecological consequences, and produced progressively cooler and somewhat drier conditions in the QTP, following rapid uplift of mountains throughout the 3.6–2.5 Ma interval.

The genera *Gymnocypris, Herzensteinia, and Schizopygosis* are the most specialized group within schizothoracines; they are also the only cyprinids that could survive in endorheic drainages of the central CTP. Given the close affinity of this highly specialized group for the severe cold‐water environments, the initial rapid radiation of them is more likely to be a response to climatic cooling, and drying following mountains uplift during the late Pliocene transition.

The divergence time between subclade NET‐E and subclade NET‐N, largely restricted to northeastern QTP, was tentatively dated back to 0.87 Ma (95% HPD: 0.56–1.11 Ma). This cladogenetic event might be associated to violent uplift of Kunlun Mountains Pass area during early‐middle Pleistocene (Kunlun‐Yellow River Tectonic Movement, 1.1–0.7 Ma, Cui et al. 1998). This tectonic movement led to a large‐scale uplifting and fault depression in northern QTP. Since then, the QTP entered cryosphere, and caused the appearance of the maximum glaciation in QTP during the Quaternary. We propose that this tectonic event caused the split the between the upper Yangtze River and drainages in the northern QTP (e.g., the upper Yellow River, Tsaidam Basin).

Within the central and southeastern Tibetan group (clade CT), the split was tentatively estimated to the Middle Pleistocene (*c*. 0.69 Ma, 95% HPD: 0.45–0.87 Ma). This cladogenetic event was also widely observed at a global scale. For example, divergences among *Telestes souffia* subspecies range across Europe occurred between 0.6–1.0 Ma (Dubut et al. 2012). In the Southern Hemisphere, two cold‐water congeneric fish species in Patagonia, *Galaxias maculatus* and *G. platei*, each experienced severe genetic bottlenecks during c. 1.1–0.6 Ma (Zemlak et al. 2011). Although estimations presented show high variation, the break coincides with the largest glaciation (Wangkun Glaciation or Naynayxungla Glaciation) in the QTP. The Naynayxungla Glaciation was probably most extensive dated glaciation throughout Tibet and the Himalaya (*c*. 0.8–0.6 Ma, according to MIS 18–16). The area of the most extensive glacier probably exceeded more than 18 times the present extent, and reached 1.7 million km^2^, which accounted for more than 60% area of the QTP (Cui et al. 1998). In the deep‐sea core record, stage 16.2 corresponds to particularly low sea level and was probably extremely cold (EPICA community members 2004). The extensive glacier and extreme cold probably caused *Schizopygosis* to descend from high altitude range (e.g., lakes in the CTP) and survive in refugia located in the exorheic rivers (e.g., the upper Salween and Tsangpo). They recolonized the formerly glaciated areas in the CTP during the followed the Günz‐Mindel (Pre‐Illinoian Cromerian) interglacial (MIS 13–15). MIS 13 was recognized as the warmest interglacial of the past 0.8 Ma in the QTP (Chen et al. 1999; Guo et al. 2009). The extreme intensity of ASM brought more precipitation associated with the glacier meltwater enhancement, which may have resulted in a high lake level in CTP, and prompted to range expansion of *Schizopygosis*.

In conclusion, our data provide first the phylogenetic pattern and demographic histories of the schizothoracine fishes, and their associated palaeoclimatic history during the Quaternary in the Changtang Plateau. Although a single mtDNA cyt *b* sequence was used, we were able to establish a clear link between the evolutionary history of the schizothoracines and the palaeoclimatic events concerning the Pleistocene glacial, lake expansion and Asia summer monsoon in the central Tibetan Plateau. The molecular evidences support the hypotheses that the Tibetan glaciations gradually diminished since the middle Pleistocene and lake expand driven by summer monsoon rainfall. Our results also suggest that the climatic shifts during the LPT and MPT at global level had a substantial impact on the genetic differentiation in the schizothoracines. However, more biogeographical analyses are needed to better understand the way in which palaeogeographical and palaeoclimatic events shaped the distribution and phylogeographical patterns of organisms in the area. It is, therefore, necessary to examine more taxa (e.g., *Tryplophysa*) and using more DNA markers in future.

## Conflict of Interest

None declared.

## Supporting information


**Appendix S1** Geographical distribution and GenBank accession numbers of haplotypes.

**Appendix S2** Statistical 95% parsimony networks ET and WT generated by TCS.

**Appendix S3** Bayesian Skyline plots showing estimates of the effective population size through time.
