# Supplementary figures and images for: Comparative phylogeography and evolutionary history of schizothoracine fishes in the Changtang Plateau and their implications for the lake level and Pleistocene climate fluctuations
Source: Ecol Evol. 2016 Jan 10;6(3):656–74. doi: 10.1002/ece3.1890 (PMC4739559; doi:10.1002/ece3.1890)

a

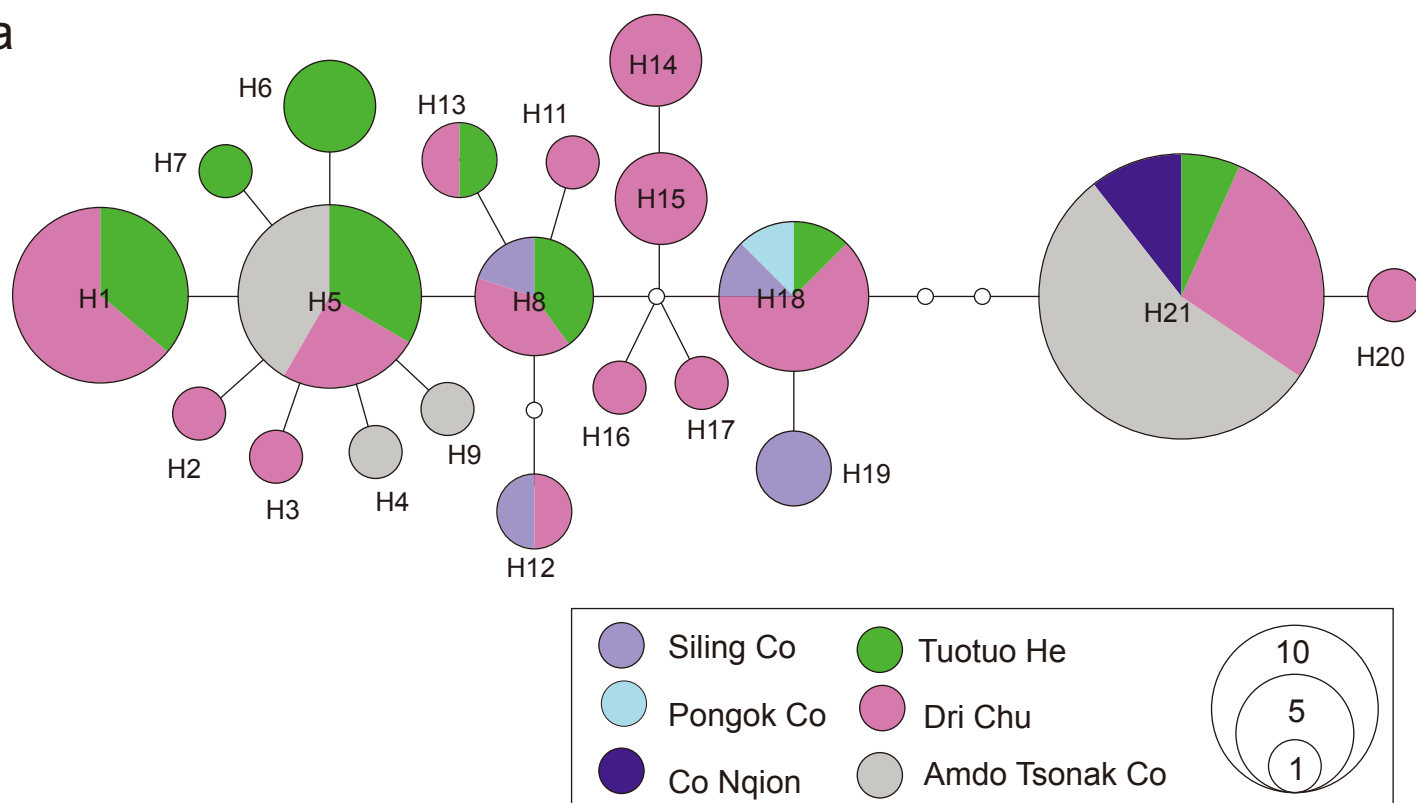

b

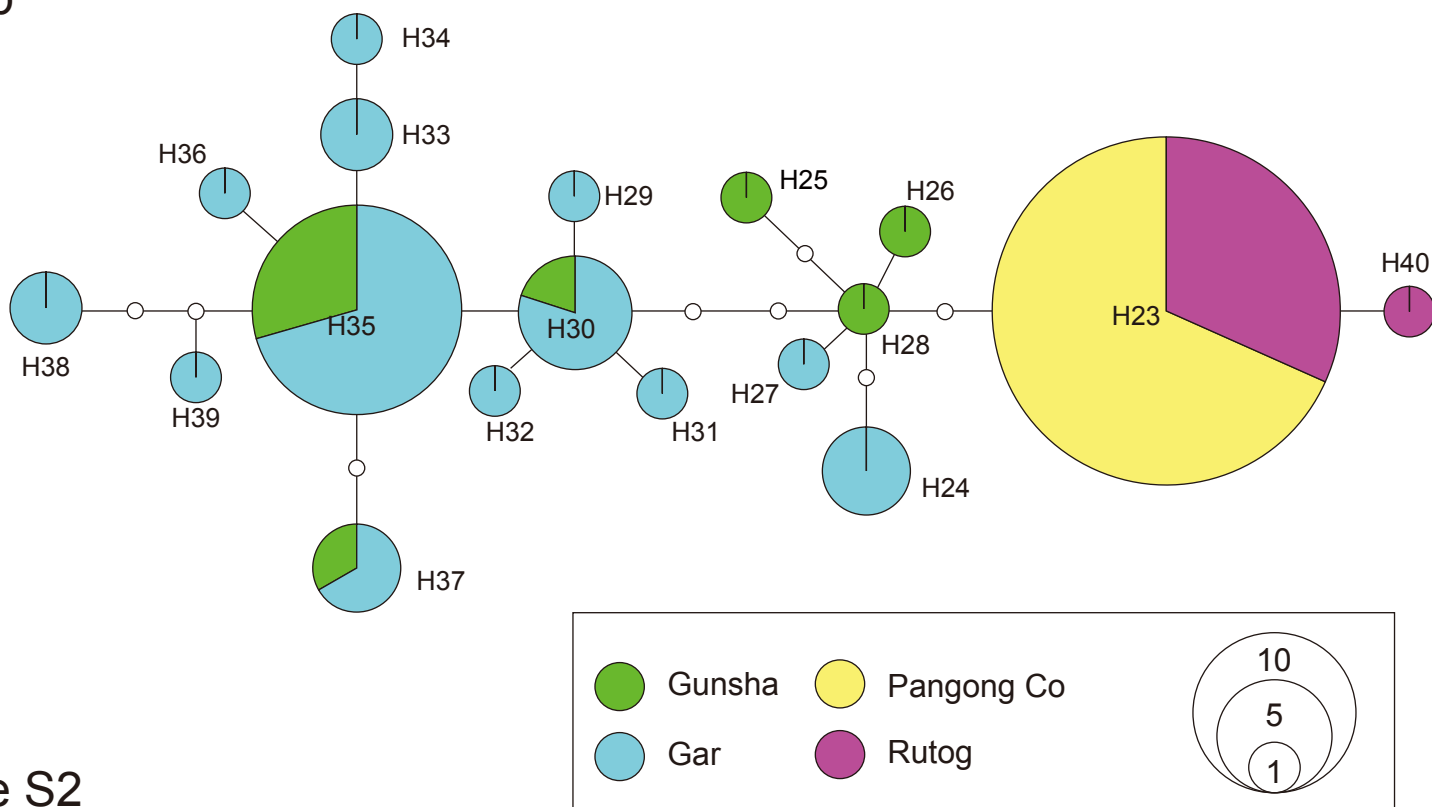

Figure S2

Supplement: Supplementary file 2 — Appendix S2 Statistical 95% parsimony networks ET and WT generated by TCS. [file ECE3-6-656-s002.pdf]

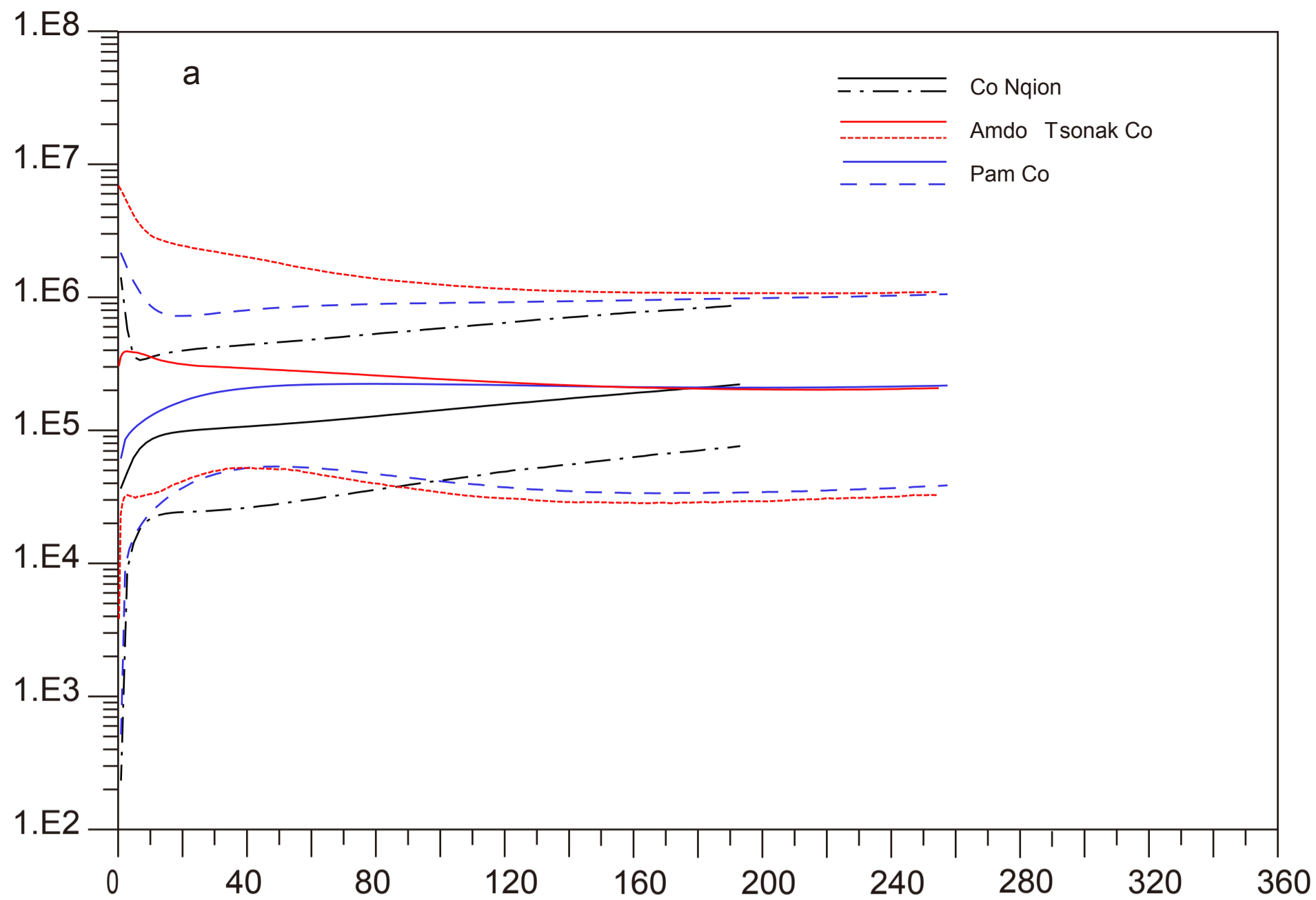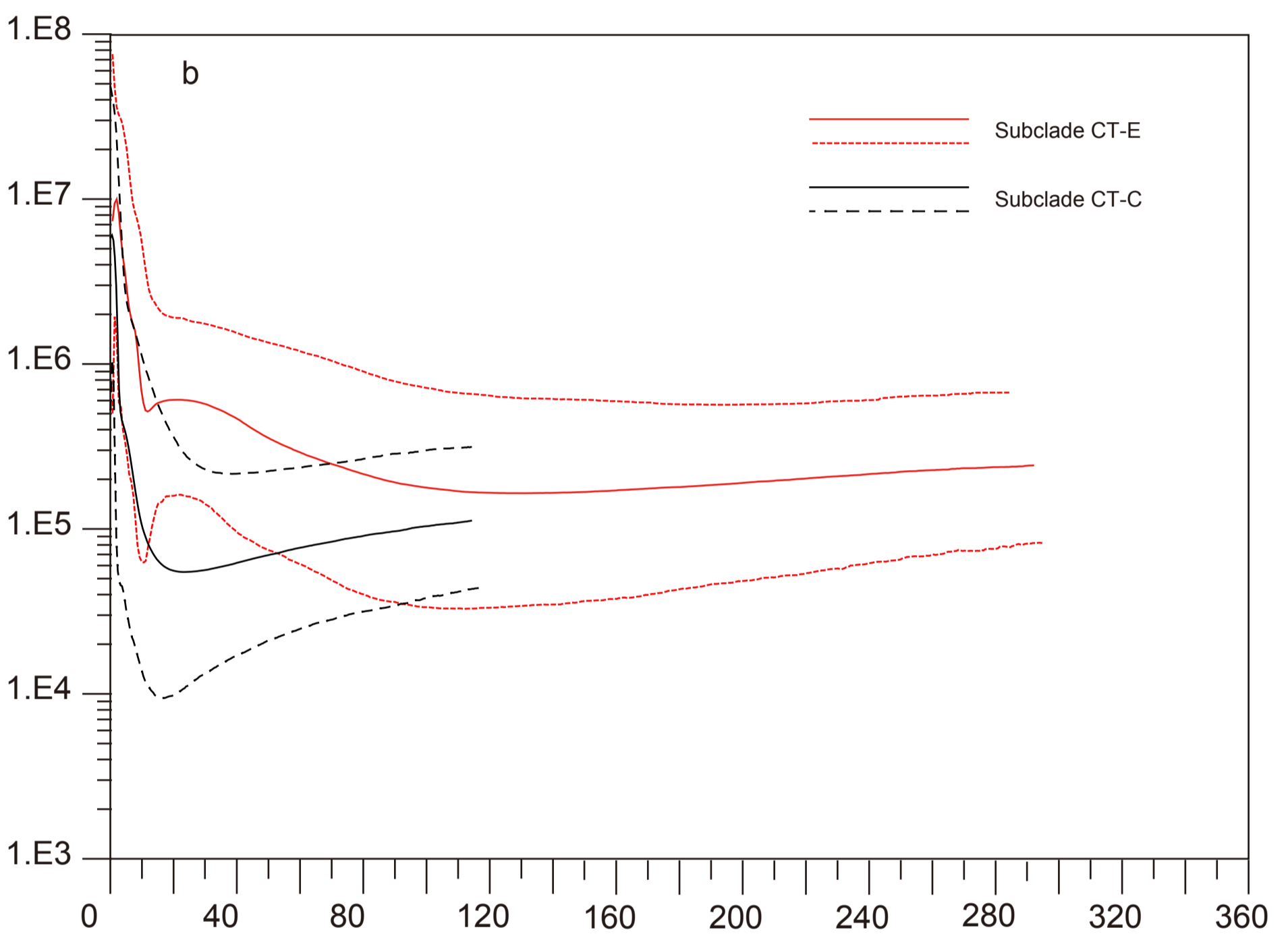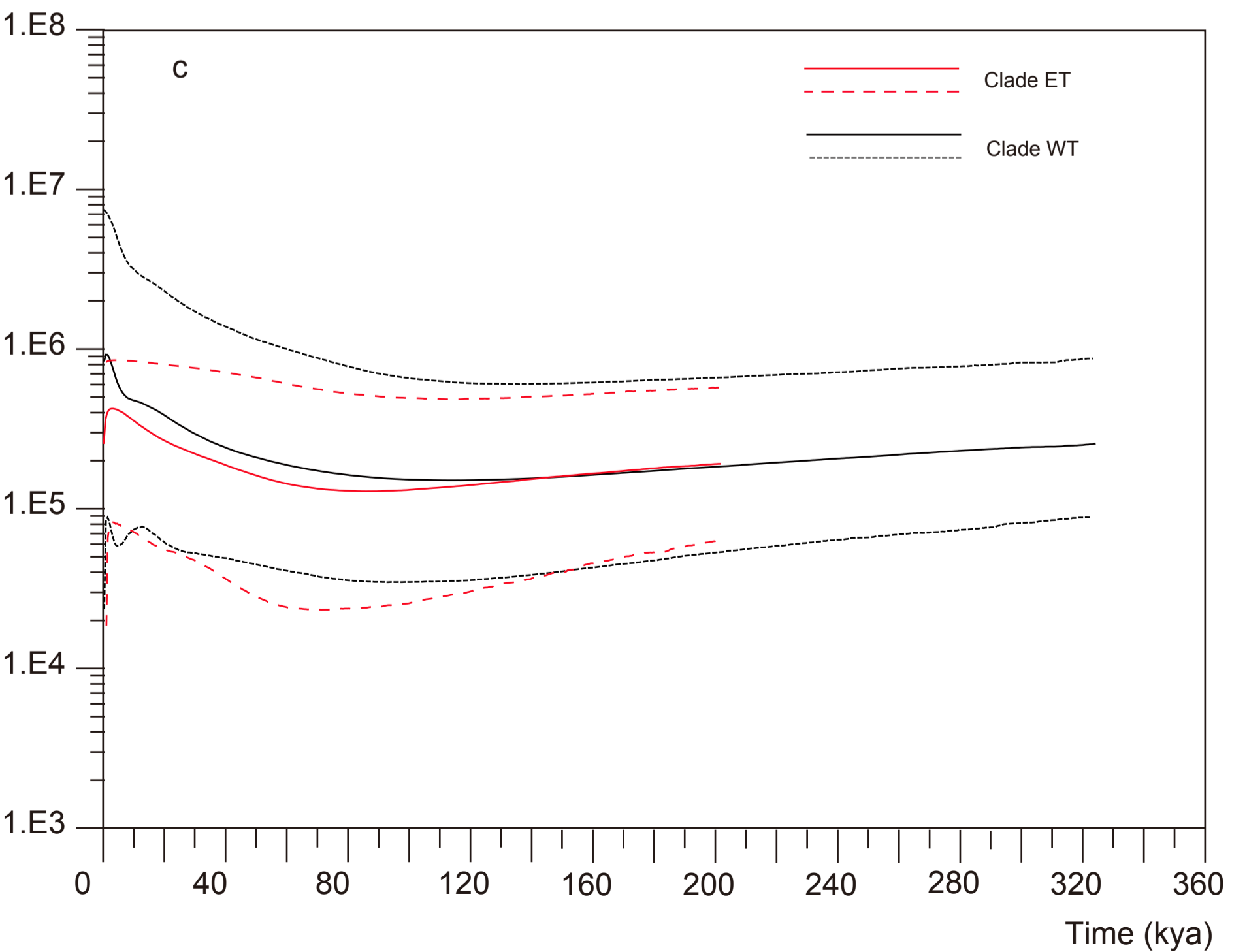

Supplement: Supplementary file 3 — Appendix S3 Bayesian Skyline plots showing estimates of the effective population size through time. [file ECE3-6-656-s003.pdf]
